# Supplementary material for: Ultralong-living magnons in the quantum limit
Source: Sci Adv. 2026 May 1;12(18):eaee2344. doi: 10.1126/sciadv.aee2344 (PMC13134583; doi:10.1126/sciadv.aee2344)
Supplement: Supplementary file 1 — Supplementary Text Figs. S1 to S3 Table S1 References [file sciadv.aee2344_sm.pdf]

Science Advances
AAAS

Supplementary Materials for

Ultralong-living magnons in the quantum limit

Rostyslav O. Serha *et al.*

Corresponding author: Rostyslav O. Serha, rostyslav.serha@univie.ac.at;
Andrii V. Chumak, andrii.chumak@univie.ac.at; Dmytro A. Bozhko, dbozhko@uccs.edu

Sci. Adv. **12**, eaee2344 (2026)
DOI: 10.1126/sciadv.aee2344

This PDF file includes:

Supplementary Text
Figs. S1 to S3
Table S1
References

Supplementary

Sample mounting and thermalization

The YIG sphere was glued to the glass spacer using GE varnish, which is commonly employed in cryogenic experiments due to its relatively good thermal conductivity at low temperatures. The glass spacer itself was anchored by GE varnish to the copper sample holder, providing a continuous thermal path from the sphere through the glass spacer to the cold stage of the dilution refrigerator. After any experimental action that could potentially heat the sample, such as magnetic-field ramps or warming above the base temperature, sufficient time (up to several hours) was allowed for the sphere to re-thermalize before measurements were resumed. Proper thermalization is reflected in the reproducible saturation behavior of the magnon lifetime and in the stability of the parametric excitation threshold.

At the lowest temperatures, the parametric threshold was measured using extremely low microwave powers, with the highest applied power for reaching threshold being approximately -50 dBm, corresponding to about 10 nW. This power level is several orders of magnitude smaller than the available cooling power of the dilution refrigerator, which is 14 μW in our setup. Therefore, assuming adequate thermal anchoring and sufficient waiting time for rethermalization after each measurement, the temperature of the sample can be considered identical to the temperature measured by the sensor at the sample holder.

There is a small heating effect of the CPW by the microwave signal passing through. It is small since the strip is impedance-matched commercial CPW with the loss typically below 1 dB, and it is well anchored to the copper holder. Besides, the signal line of the stripline is anchored via the 0 dB attenuator at different temperature levels, including the lowest one. Thus, we observe heating of the sensor's temperature only if the applied microwave power is above -20 dBm (at the lowest temperature 30 mK). At higher temperatures, larger powers can be used without heating the system.

To further verify that the measurement procedure itself does not induce sample heating due to magnetization excitation, control measurements were performed on sphere 1 at 30 mK using different intermediate frequency bandwidths (IFBW) ranging from 30 kHz down to 30 Hz. Changing the IFBW modifies the sweep time, and thus the total time spent probing the FMR region, by approximately three orders of magnitude. If microwave excitation were to heat the sample, a reduced IFBW—corresponding to longer measurement times at resonance—would result in an increased parametric threshold. However, no change in the threshold power was observed for any IFBW. This demonstrates that the microwave power levels used (−50 dBm and below) do not cause measurable self-heating of the sphere, even at 30 mK. We therefore conclude that, for all measurements reported, the sample remained in thermal equilibrium with the cryostat and that the temperatures recorded by the sensors accurately reflect the sample temperature.

Three-magnon scattering at 3.87 GHz

In addition to the secondary magnon lifetime results presented in Fig. 2 for measurements at an FMR frequency of 3.17 GHz, we provide supplementary measurements at a higher frequency of 3.87 GHz in fig. S3 to further support the conclusions in the main text. Measurements at 3.87 GHz could not be performed at room temperature (RT) due to the lower saturation magnetization M_s of YIG at higher temperatures, which makes the three-magnon scattering process

inaccessible at this frequency. However, for consistency and ease of comparison, fig. S3 maintains the same scale as Fig. 2.

Figure. S3A shows the parametric power threshold P_{thr} as a function of temperature and fig. S3B shows the corresponding threshold rf field b_{thr}, plotted on a logarithmic x-axis. Among the three spheres, sphere 3 (black) exhibits the lowest threshold power and rf field across the entire temperature range, with b_{thr} decreasing as the temperature drops and saturating below 600 mK. The behavior of sphere 2 (yellow) and sphere 1 (blue) follows a similar trend, but both show significantly larger threshold rf fields compared to sphere 3. Notably, sphere 1 shows a steeper decrease in b_{thr} resulting in smaller threshold rf fields compared to sphere 2 at temperatures below 1 K. This behavior can be understood by examining the FMR linewidths presented in Fig. 2A. As shown, the linewidth of sphere 1 decreases steeply due to the freezing out of rare-earth impurities, making it smaller than that of sphere 2 within the same temperature range.

Overall, the threshold rf fields for all spheres are larger at an FMR frequency of 3.87 GHz compared to the 3.17 GHz measurements, which is consistent with the higher magnon frequency requiring stronger driving fields for instability.

As described in the main manuscript, the lifetimes of secondary magnons are extracted from the threshold rf field values b_{thr}, and these are shown in fig. S3D. The behavior of DEMs with a frequency of 1.94 GHz (corresponding to the 3.87 GHz FMR frequency) closely resembles the results for 1.59 GHz magnons presented in Fig. 2B. sphere 1 exhibits the shortest magnon lifetimes, followed by sphere 2, while sphere 3 shows the longest lifetimes, reaching a maximum of approximately 14 μs. The increase in magnon lifetime saturates earlier for 1.94 GHz magnons, below 400 mK, compared to the results for 1.59 GHz magnons (around 100 mK). One possible explanation for the observed difference in saturation behavior is that, at higher frequencies, the thermal baths of magnons and phonons involved in the scattering processes described in the main article freeze out at comparatively higher temperatures.

The magnon lifetimes at 1.94 GHz are shorter than those at 1.59 GHz for all YIG spheres. Specifically, the lifetimes are reduced by approximately 12% for sphere 1, 15% for sphere 2 and 20% for sphere 3. These results confirm that secondary magnon lifetimes decrease with increasing magnon frequency.

<u>Magnon relaxation channels</u>

Although these are the first reported measurements of $k \neq 0$ magnon lifetimes in the quantum limit, relaxation mechanisms contributing to magnon damping have been studied previously down to a Kelvin range of temperatures (31, 32, 54, 57, 83-86). The rather high election band gap of 2.8 eV of YIG (87) precludes magnon-electron relaxation processes (63). The dominant mechanism defining the FMR linewidth ΔH_0 is elastic two-magnon scattering, which is temperature-independent. As most of the defects are associated with surface roughness, the best FMR linewidths could be achieved for samples with the highest volume-to-surface ratio – spheres. As a result, ΔH_0 remains nearly constant with temperature (see table S1) for the purest YIG sphere 3, where magnon-ion relaxation is greatly reduced.

On the contrary, for the short-wavelength magnons, we observe a pronounced temperature dependence of the threshold power for three-magnon splitting, which suggests that their relaxation, often referred to by the linewidth ΔH_k, is not dominated by two-magnon scattering (88) and therefore is governed by other processes. These processes include intrinsic effects of magnon-magnon (also known as spin-spin) (89, 90) or magnon-phonon (or spin-lattice) (59, 91) interactions, as well as extrinsic effects of coupling to magnetic moments of impurities (12, 72).

Three-magnon splitting is forbidden for the secondary magnons due to their location close to the bottom of the spin-wave spectrum (see Fig. 1B). Other nonlinear relaxation processes such as three-magnon confluence and four-magnon scattering are exponentially ineffective, as they require large magnon populations (either thermal or stimulated) spectrally close to the magnons in question, which is not the case for our experiments done in the $T \rightarrow 0$ limit. This statement is further supported by the fact that the damping behavior below 100 mK experiences saturation for all samples. This temperature is associated with the thermal energy of $k_B T \approx 2$ GHz, which is close to the experimentally measured frequency of 1.59 GHz, and, therefore, the magnon (as well as phonon) population below this temperature is strongly suppressed.

The direct magnon-phonon interaction processes, including the so-called Kasuya-LeCraw mechanism (92) available for an ideal crystal lattice and only small wavevectors, scale well with temperature as the population of the phononic bath is effectively suppressed in the $T \rightarrow 0$ limit (93). When magnon's group velocity is higher than the phonon's one, magnons can scatter into another magnon and a phonon – the so-called Cherenkov process. However, this effect is also suppressed for short-wavelength magnons with moderate frequencies. But despite the impression that all the damping mechanisms are becoming suppressed, the observed magnon relaxation here does not vanish at $T \rightarrow 0$, as predicted theoretically for ultra-pure YIG. In our case, the residual magnon damping can still be attributed to the intrinsic magnon interaction with the low-frequency portion of the phonon bath. Since phononic spectrum does not have a gap, this contribution will always be present at finite temperature. However, this contribution is intrinsic and therefore should be equal for all the studied spheres, which is clearly not the case in our findings – see Fig. 3B. That gives a strong hint that the main contributor to the residual damping is coming from impurities.

Despite the ultra-high purity of sphere 3, we cannot guarantee that the residual impurity concentration was zero. Such solitary paramagnetic impurities, which include either rare-earth elements or Pt^{4+}, Si^{4+}, Fe^{2+}, and Fe^{4+} originated from the crystal growth process (92), directly interact with magnons through their fluctuating magnetic moment and are called two-level-fluctuators (TLF), as proposed in Refs. (12, 72). Their influence strongly depends on their own relaxation frequency, which scales with temperature and should vanish at $T = 0$ (54) but apparently in our case, that was not yet achieved. The design of a particular experiment to quantify TLFs' influence on short-wavelength magnons constitutes a fundamentally interesting problem, which spans outside the scope of the current work. However, our findings clearly show that 18 μs, as reported here, is not the fundamental limit of the lifetime of this type of boson and that it can be significantly increased further by optimizing the fabrication technology.

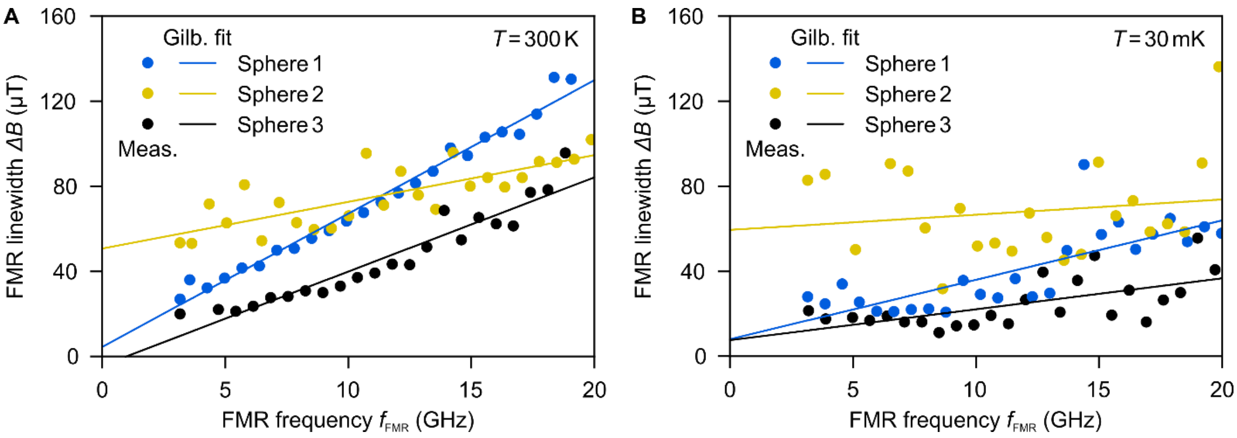

Fig. S1.

FMR linewidth ΔB as a function of the FMR frequency f_{FMR}. The dots represent the measured linewidths for three different YIG spheres: sphere 1 (blue), sphere 2 (yellow), and sphere 3 (black), while the solid lines show the corresponding linear Gilbert fit. Panel A displays measurements taken at 300 K, and B shows measurements at 30 mK. From the measurements it becomes evident that sphere 3 has the lowest damping parameters for both temperatures. While sphere 2 has a smaller slope, corresponding to a lower Gilbert damping parameter α, it also has a higher ΔB_0.

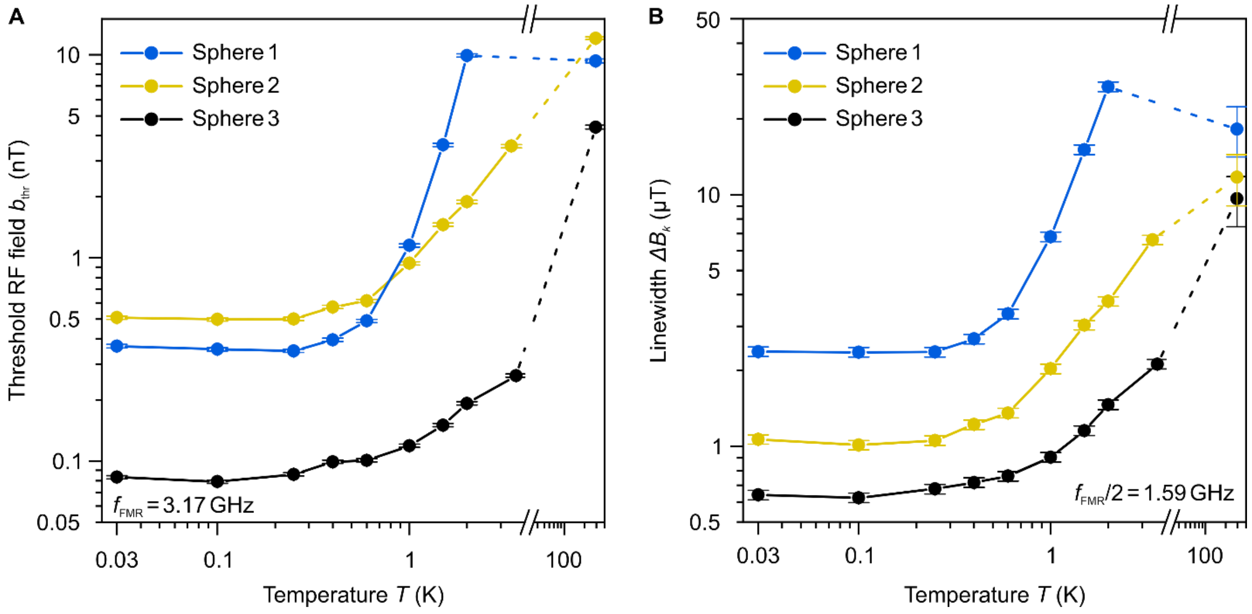

Fig. S2.

Threshold rf field b_{thr} and linewidth ΔB_k vs temperature T. A Threshold rf field b_{thr} as a function of temperature on a logarithmic x-axis for three different YIG spheres at the FMR frequency of 3.17 GHz. **B** Linewidth of secondary magnons ΔB_k with the frequency half of the FMR frequency vs the temperature T on a logarithmic x-axis for three different YIG spheres. These figures complement Fig. 3 from the main manuscript.

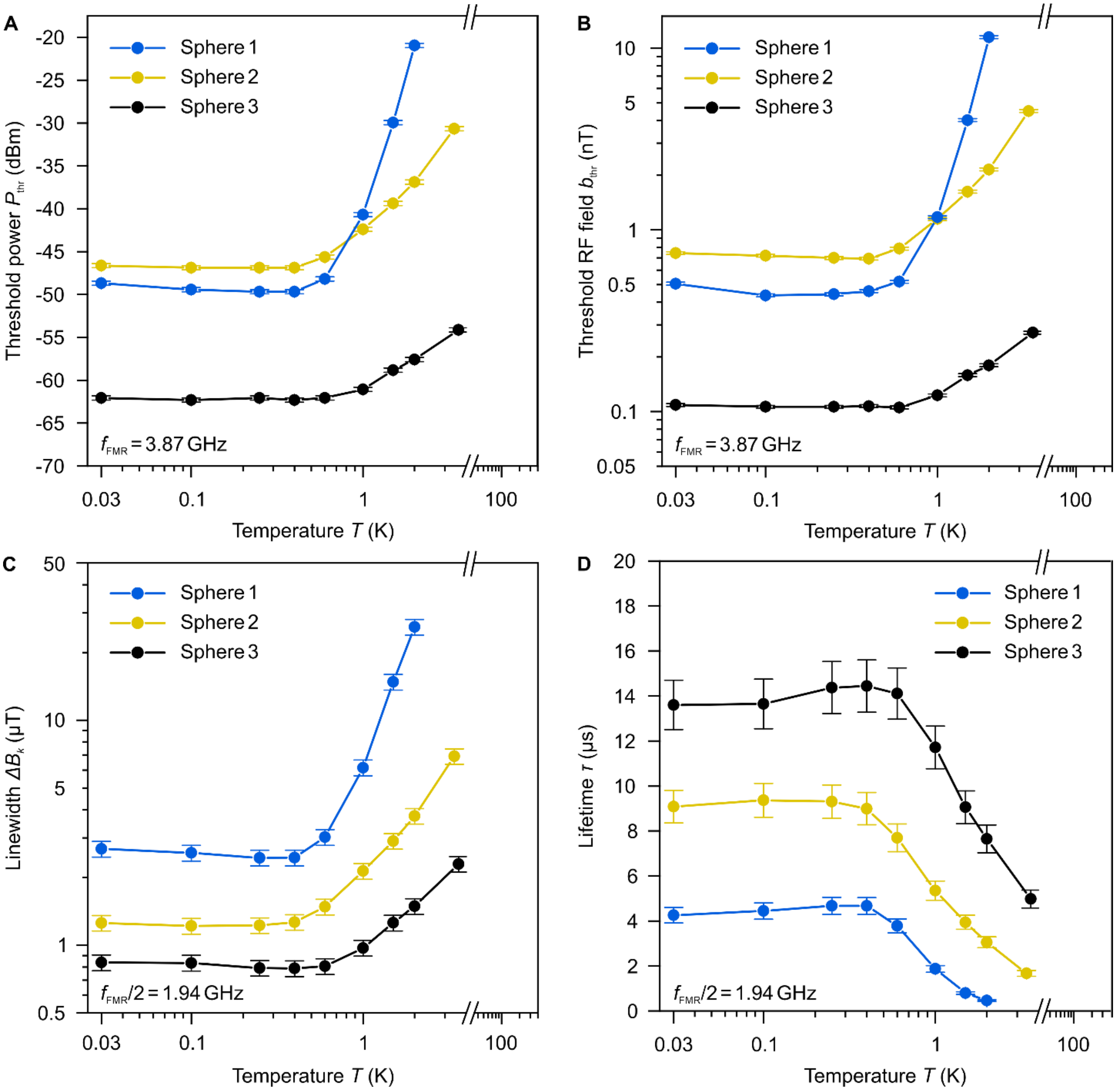

Fig. S3.

Measurement of secondary magnons for f_{FMR} = 3.87 GHz vs temperature. A Experimental measurements of the parametric power threshold P_{thr} of three different YIG spheres as a function of the temperature T plotted in a logarithmic x-axis for a FMR frequency of 3.87 GHz. **B** Threshold rf field b_{thr} as a function of temperature on a logarithmic x-axis for three different YIG spheres at the FMR frequency of 3.87 GHz. **C** Linewidth of secondary magnons ΔB_k with the frequency half of the FMR frequency vs the temperature T on a logarithmic x-axis for three different YIG spheres. **D** Lifetime τ of secondary magnons vs the temperature T on a logarithmic x-axis for three different YIG spheres. It was not possible to take measurements at 3.87 GHz at RT because the lower saturation magnetization M_s of YIG at higher temperatures renders the three-magnon scattering process inaccessible at this frequency.

Table S1.

Gilbert fit parameters. Gilbert damping α and inhomogeneous linewidth broadening ΔB_0 for three different YIG Spheres and two distinct temperatures, 300 K and 30 mK from fig. S1.

**Sample:**	**Gilbert damping α (10^{-5})**		**Inhomogeneous linewidth broadening ΔB_0 (µT)**	
**Temperature**	**@ 300 K**	**@ 30 mK**	**@ 300 K**	**@ 30 mK**
**Sphere 1**	**8.78 ± 0.26**	**3.92 ± 0.67**	**4.45 ± 2.23**	**8.08 ± 6.01**
**Sphere 2**	**3.08 ± 0.50**	**1.01 ± 1.34**	**50.72 ± 4.54**	**59.46 ± 12.47**
**Sphere 3**	**6.21± 0.44**	**2.05 ± 0.54**	**-4.35 ± 3.86**	**7.61 ± 4.85**

REFERENCES

1. J. Preskill, Quantum computing in the NISQ era and beyond. *Quantum* **2**, 79 (2018).

2. M. H. Devoret, R. J. Schoelkopf, Superconducting circuits for quantum information: An outlook. *Science* **339**, 1169–1174 (2013).

3. A. V. Kuhlmann, J. Houel, A. Ludwig, L. Greuter, D. Reuter, A. D. Wieck, M. Poggio, R. J. Warburton, Charge noise and spin noise in a semiconductor quantum device. *Nat. Phys.* **9**, 570–575 (2013).

4. S. Knauer, J. P. Hadden, J. G. Rarity, In-situ measurements of fabrication induced strain in diamond photonic-structures using intrinsic colour centres. *npj Quantum Inf.* **6**, 50 (2020).

5. Y.-C. Chen, P. S. Salter, S. Knauer, L. Weng, A. C. Frangeskou, C. J. Stephen, S. N. Ishmael, P. R. Dolan, S. Johnson, B. L. Green, G. W. Morley, M. E. Newton, J. G. Rarity, M. J. Booth, J. M. Smith, Laser writing of coherent colour centres in diamond. *Nat. Photonics* **11**, 77–80 (2017).

6. A. A. Gentile, B. Flynn, S. Knauer, N. Wiebe, S. Paesani, C. E. Granade, J. G. Rarity, R. Santagati, A. Laing, Learning models of quantum systems from experiments. *Nat. Phys.* **17**, 837–843 (2021).

7. J. Bochmann, A. Vainsencher, D. D. Awschalom, A. N. Cleland, Nanomechanical coupling between microwave and optical photons. *Nat. Phys.* **9**, 712–716 (2013).

8. H. Qiao, Z. Wang, G. Andersson, A. Anferov, C. R. Conner, Y. J. Joshi, S. Li, J. M. Miller, X. Wu, H. Yan, L. Jiang, A. N. Cleland, Acoustic phonon phase gates with number-resolving phonon detection. *Nat. Phys.* **21**, 1801–1805 (2025).

9. H. Wang, A. Kumar, S. Dai, X. Lin, Z. Jacob, S.-H. Oh, V. Menon, E. Narimanov, Y. D. Kim, J.-P. Wang, P. Avouris, L. Martin Moreno, J. Caldwell, T. Low, Planar hyperbolic polaritons in 2D van der Waals materials. *Nat. Commun.* **15**, 69 (2024).

10. P. Pirro, V. I. Vasyuchka, A. A. Serga, B. Hillebrands, Advances in coherent magnonics. *Nat. Rev. Mater.* **6**, 1114–1135 (2021).

11. M. Harder, B. M. Yao, Y. S. Gui, C.-M. Hu, Coherent and dissipative cavity magnonics. *J. Appl. Phys.* **129**, 201101 (2021).

12. S. Kosen, A. F. van Loo, D. A. Bozhko, L. Mihalceanu, A. D. Karenowska, Microwave magnon damping in YIG films at millikelvin temperatures. *APL Mater.* **7**, 101120 (2019).

13. P. V. Klimov, A. L. Falk, D. J. Christle, V. V. Dobrovitski, D. D. Awschalom, Quantum entanglement at ambient conditions in a macroscopic solid-state spin ensemble. *Sci. Adv.* **1**, e1501015 (2015).

14. D. D. Awschalom, C. R. Du, R. He, F. J. Heremans, A. Hoffmann, J. Hou, H. Kurebayashi, Y. Li, L. Liu, V. Novosad, J. Sklenar, S. E. Sullivan, D. Sun, H. Tang, V. Tyberkevych, C. Trevillian, A. W. Tsen, L. R. Weiss, W. Zhang, X. Zhang, L. Zhao, C. W. Zollitsch, Quantum engineering with hybrid magnonic systems and materials (Invited Paper). *IEEE Trans. Quantum Eng.* **2**, 1–36 (2021).

15. A. V. Kondrashov, A. B. Ustinov, Self-generation of Möbius solitons and chaotic waveforms in magnonic-optoelectronic oscillators under simultaneous action of optic and magnonic nonlinearities. *J. Appl. Phys.* **132**, 173907 (2022).

16. A. A. Serga, A. V. Chumak, B. Hillebrands, YIG magnonics. *J. Phys. D Appl. Phys.* **43**, 264002 (2010).

17. C. L. Ordóñez-Romero, M. A. Cherkasskii, N. Qureshi, B. A. Kalinikos, C. E. Patton, Direct Brillouin light scattering observation of dark spin-wave envelope solitons in magnetic films. *Phys. Rev. B* **87**, 174430 (2013).

18. V. I. Vasyuchka, G. A. Melkov, A. N. Slavin, A. V. Chumak, V. A. Moiseienko, B. Hillebrands, Non-resonant wave front reversal of spin waves used for microwave signal processing. *J. Phys. D Appl. Phys.* **43**, 325001 (2010).

19. M. Kostylev, Non-reciprocity of dipole-exchange spin waves in thin ferromagnetic films. *J. Appl. Phys.* **113**, 053907 (2013).

20. R. Verba, V. Tiberkevich, A. Slavin, Wide-band nonreciprocity of surface acoustic waves induced by magnetoelastic coupling with a synthetic antiferromagnet. *Phys. Rev. Appl.* **12**, 054061 (2019).

21. Q. Wang, P. Pirro, R. Verba, A. Slavin, B. Hillebrands, A. V. Chumak, Reconfigurable nanoscale spin-wave directional coupler. *Sci. Adv.* **4**, e1701517 (2018).

22. M. Mohseni, R. Verba, T. Brächer, Q. Wang, D. A. Bozhko, B. Hillebrands, P. Pirro, Backscattering immunity of dipole-exchange magnetostatic surface spin waves. *Phys. Rev. Lett.* **122**, 197201 (2019).

23. M. Mohseni, M. Kewenig, R. Verba, Q. Wang, M. Schneider, B. Heinz, F. Kohl, C. Dubs, B. Lägel, A. A. Serga, B. Hillebrands, A. V. Chumak, P. Pirro, Parametric generation of propagating spin waves in ultrathin yttrium iron garnet waveguides. *Phys. Status Solidi RRL* **14**, 2000011 (2020).

24. D. A. Bozhko, H. Y. Musiienko-Shmarova, V. S. Tiberkevich, A. N. Slavin, I. I. Syvorotka, B. Hillebrands, A. A. Serga, Unconventional spin currents in magnetic films. *Phys. Rev. Res.* **2**, 023324 (2020).

25. D. A. Bozhko, A. J. E. Kreil, H. Y. Musiienko-Shmarova, A. A. Serga, A. Pomyalov, V. S. L'vov, B. Hillebrands, Bogoliubov waves and distant transport of magnon condensate at room temperature. *Nat. Commun.* **10**, 2460 (2019).

26. D. A. Bozhko, A. A. Serga, P. Clausen, V. I. Vasyuchka, F. Heussner, G. A. Melkov, A. Pomyalov, V. S. L'vov, B. Hillebrands, Supercurrent in a room-temperature Bose–Einstein magnon condensate. *Nat. Phys.* **12**, 1057–1062 (2016).

27. M. Schneider, T. Brächer, D. Breitbach, V. Lauer, P. Pirro, D. A. Bozhko, H. Y. Musiienko-Shmarova, B. Heinz, Q. Wang, T. Meyer, F. Heussner, S. Keller, E. T. Papaioannou, B. Lägel, T. Löber, C. Dubs, A. N. Slavin, V. S. Tiberkevich, A. A. Serga, B. Hillebrands, A. V.

Chumak, Bose–Einstein condensation of quasiparticles by rapid cooling. *Nat. Nanotechnol.* **15**, 457–461 (2020).

28. Q. Wang, R. Verba, B. Heinz, M. Schneider, O. Wojewoda, K. Davídková, K. Levchenko, C. Dubs, N. J. Mauser, M. Urbánek, P. Pirro, A. V. Chumak, Deeply nonlinear excitation of self-normalized short spin waves. *Sci. Adv.* **9**, eadg4609 (2023).

29. H. Y. Yuan, Y. Cao, A. Kamra, R. A. Duine, P. Yan, Quantum magnonics: When magnon spintronics meets quantum information science. *Phys. Rep.* **965**, 1–74 (2022).

30. A. V. Chumak, P. Kabos, M. Wu, C. Abert, C. Adelmann, A. O. Adeyeye, J. Akerman, F. G. Aliev, A. Anane, A. Awad, C. H. Back, A. Barman, G. E. W. Bauer, M. Becherer, E. N. Beginin, V. A. S. V. Bittencourt, Y. M. Blanter, P. Bortolotti, I. Boventer, D. A. Bozhko, S. A. Bunyaev, J. J. Carmiggelt, R. R. Cheenikundil, F. Ciubotaru, S. Cotofana, G. Csaba, O. V. Dobrovolskiy, C. Dubs, M. Elyasi, K. G. Fripp, H. Fulara, I. A. Golovchanskiy, C. Gonzalez-Ballestero, P. Graczyk, D. Grundler, P. Gruszecki, G. Gubbiotti, K. Guslienko, A. Haldar, S. Hamdioui, R. Hertel, B. Hillebrands, T. Hioki, A. Houshang, C.-M. Hu, H. Huebl, M. Huth, E. Iacocca, M. B. Jungfleisch, G. N. Kakazei, A. Khitun, R. Khymyn, T. Kikkawa, M. Klaui, O. Klein, J. W. Klos, S. Knauer, S. Koraltan, M. Kostylev, M. Krawczyk, I. N. Krivorotov, V. V. Kruglyak, D. Lachance-Quirion, S. Ladak, R. Lebrun, Y. Li, M. Lindner, R. Macedo, S. Mayr, G. A. Melkov, S. Mieszczak, Y. Nakamura, H. T. Nembach, A. A. Nikitin, S. A. Nikitov, V. Novosad, J. A. Otalora, Y. Otani, A. Papp, B. Pigeau, P. Pirro, W. Porod, F. Porrati, H. Qin, B. Rana, T. Reimann, F. Riente, O. Romero-Isart, A. Ross, A. V. Sadovnikov, A. R. Safin, E. Saitoh, G. Schmidt, H. Schultheiss, K. Schultheiss, A. A. Serga, S. Sharma, J. M. Shaw, D. Suess, O. Surzhenko, K. Szulc, T. Taniguchi, M. Urbanek, K. Usami, A. B. Ustinov, T. van der Sar, S. van Dijken, V. I. Vasyuchka, R. Verba, S. V. Kusminskiy, Q. Wang, M. Weides, M. Weiler, S. Wintz, S. P. Wolski, X. Zhang, Advances in magnetics roadmap on spin-wave computing. *IEEE Trans. Magn.* **58**, 0800172 (2022).

31. P. G. Baity, D. A. Bozhko, R. Macêdo, W. Smith, R. C. Holland, S. Danilin, V. Seferai, J. Barbosa, R. R. Peroor, S. Goldman, U. Nasti, J. Paul, R. H. Hadfield, S. McVitie, M. Weides, Strong magnon–photon coupling with chip-integrated YIG in the zero-temperature limit. *Appl. Phys. Lett.* **119**, 033502 (2021).

32. R. Macêdo, R. C. Holland, P. G. Baity, L. J. Mclellan, K. L. Livesey, R. L. Stamps, M. P. Weides, D. A. Bozhko, Electromagnetic approach to cavity spintronics. *Phys. Rev. Appl.* **15**, 024065 (2021).

33. Y. Tabuchi, S. Ishino, A. Noguchi, T. Ishikawa, R. Yamazaki, K. Usami, Y. Nakamura, Coherent coupling between a ferromagnetic magnon and a superconducting qubit. *Science* **349**, 405–408 (2015).

34. D. Lachance-Quirion, S. P. Wolski, Y. Tabuchi, S. Kono, K. Usami, Y. Nakamura, Entanglement-based single-shot detection of a single magnon with a superconducting qubit. *Science* **367**, 425–428 (2020).

35. Y. Li, W. Zhang, V. Tyberkevych, W.-K. Kwok, A. Hoffmann, V. Novosad, Hybrid magnonics: Physics, circuits, and applications for coherent information processing. *J. Appl. Phys.* **128**, 130902 (2020).

36. Y. Li, T. Polakovic, Y.-L. Wang, J. Xu, S. Lendinez, Z. Zhang, J. Ding, T. Khaire, H. Saglam, R. Divan, J. Pearson, W.-K. Kwok, Z. Xiao, V. Novosad, A. Hoffmann, W. Zhang, Strong coupling between magnons and microwave photons in on-chip ferromagnet-superconductor thin-film devices. *Phys. Rev. Lett.* **123**, 107701 (2019).

37. D. Xu, X.-K. Gu, Y.-C. Weng, H.-K. Li, Y.-P. Wang, S.-Y. Zhu, J. Q. You, Macroscopic Bell state between a millimeter-sized spin system and a superconducting qubit. *Quantum Sci. Technol.* **9**, 035002 (2024).

38. A. Kamra, W. Belzig, A. Brataas, Magnon-squeezing as a niche of quantum magnonics. *Appl. Phys. Lett.* **117**, 090501 (2020).

39. Z. Haghshenasfard, M. G. Cottam, Quantum statistics and squeezing for a microwave-driven interacting magnon system. *J. Phys. Condens. Matter* **29**, 045803 (2017).

40. J. M. P. Nair, B. Flebus, Engineering long-lived entanglement through dissipation in quantum hybrid solid-state platforms. *New J. Phys.* **27**, 093501 (2025).

41. B. Zare Rameshti, S. Viola Kusminskiy, J. A. Haigh, K. Usami, D. Lachance-Quirion, Y. Nakamura, C.-M. Hu, H. X. Tang, G. E. W. Bauer, Y. M. Blanter, Cavity magnonics. *Phys. Rep.* **979**, 1–61 (2022).

42. M. Elyasi, Y. M. Blanter, G. E. W. Bauer, Resources of nonlinear cavity magnonics for quantum information. *Phys. Rev. B* **101**, 054402 (2020).

43. X. Zhang, C.-L. Zou, L. Jiang, H. X. Tang, Cavity magnomechanics. *Sci. Adv.* **2**, e1501286 (2016).

44. R. O. Serha, C. Dubs, A. V. Chumak, Magnetic materials for quantum magnonics. *APL Mater.* **14**, 09331 (2026).

45. D. Xu, X.-K. Gu, H.-K. Li, Y.-C. Weng, Y.-P. Wang, J. Li, H. Wang, S.-Y. Zhu, J. Q. You, Quantum control of a single magnon in a macroscopic spin system. *Phys. Rev. Lett.* **130**, 193603 (2023).

46. S. Knauer, K. Davídková, D. Schmoll, R. O. Serha, A. Voronov, Q. Wang, R. Verba, O. V. Dobrovolskiy, M. Lindner, T. Reimann, C. Dubs, M. Urbánek, A. V. Chumak, Propagating spin-wave spectroscopy in a liquid-phase epitaxial nanometer-thick YIG film at millikelvin temperatures. *J. Appl. Phys.* **133**, 143905 (2023).

47. R. O. Serha, C. Dubs, C. Guguschev, B. Aichner, D. Schmoll, J. Schäfer, J. Panda, M. Weiler, P. Pirro, M. Urbánek, A. V. Chumak, The ideal substrate for yttrium iron garnet films in quantum magnonics. *Commun. Mater.,* 10.1038/s43246-026-01146-5 (2026).

48. D. Schmoll, A. A. Voronov, R. O. Serha, D. Slobodianiuk, K. O. Levchenko, C. Abert, S. Knauer, D. Suess, R. Verba, A. V. Chumak, Wavenumber-dependent magnetic losses in yttrium iron garnet–gadolinium gallium garnet heterostructures at millikelvin temperatures. *Phys. Rev. B* **111**, 134428 (2025).

49. J. Bensmann, R. Schmidt, K. O. Nikolaev, D. Raskhodchikov, S. Choudhary, R. Bhardwaj, S. Taheriniya, A. Varri, S. Niehues, A. El Kadri, J. Kern, W. H. P. Pernice, S. O. Demokritov, V. E. Demidov, S. Michaelis de Vasconcellos, R. Bratschitsch, Dispersion-tunable low-loss

implanted spin-wave waveguides for large magnonic networks. *Nat. Mater.* **24**, 1920–1926 (2025).

50. V. Cherepanov, I. Kolokolov, V. L'vov, The saga of YIG: Spectra, thermodynamics, interaction and relaxation of magnons in a complex magnet. *Phys. Rep.* **229**, 81–144 (1993).

51. S. Geller, M. A. Gilleo, Structure and ferrimagnetism of yttrium and rare-earth–iron garnets. *Acta Crystallogr.* **10**, 239–239 (1957).

52. H. Huebl, C. W. Zollitsch, J. Lotze, F. Hocke, M. Greifenstein, A. Marx, R. Gross, S. T. B. Goennenwein, High cooperativity in coupled microwave resonator ferrimagnetic insulator hybrids. *Phys. Rev. Lett.* **111**, 127003 (2013).

53. X. Zhang, C.-L. Zou, L. Jiang, H. X. Tang, Strongly coupled magnons and cavity microwave photons. *Phys. Rev. Lett.* **113**, 156401 (2014).

54. C. L. Jermain, S. V. Aradhya, N. D. Reynolds, R. A. Buhrman, J. T. Brangham, M. R. Page, P. C. Hammel, F. Y. Yang, D. C. Ralph, Increased low-temperature damping in yttrium iron garnet thin films. *Phys. Rev. B* **95**, 174411 (2017).

55. S. Klingler, H. Maier-Flaig, C. Dubs, O. Surzhenko, R. Gross, H. Huebl, S. T. B. Goennenwein, M. Weiler, Gilbert damping of magnetostatic modes in a yttrium iron garnet sphere. *Appl. Phys. Lett.* **110**, 092409 (2017).

56. H. Maier-Flaig, S. Klingler, C. Dubs, O. Surzhenko, R. Gross, M. Weiler, H. Huebl, S. T. B. Goennenwein, Temperature-dependent magnetic damping of yttrium iron garnet spheres. *Phys. Rev. B* **95**, 214423 (2017).

57. L. Mihalceanu, V. I. Vasyuchka, D. A. Bozhko, T. Langner, A. Y. Nechiporuk, V. F. Romanyuk, B. Hillebrands, A. A. Serga, Temperature-dependent relaxation of dipole-exchange magnons in yttrium iron garnet films. *Phys. Rev. B* **97**, 214405 (2018).

58. T. Makiuchi, T. Hioki, H. Shimizu, K. Hoshi, M. Elyasi, K. Yamamoto, N. Yokoi, A. A. Serga, B. Hillebrands, G. E. W. Bauer, E. Saitoh, Persistent magnetic coherence in magnets. *Nat. Mater.* **23**, 627–632 (2024).

59. A. Rückriegel, P. Kopietz, D. A. Bozhko, A. A. Serga, B. Hillebrands, Magnetoelastic modes and lifetime of magnons in thin yttrium iron garnet films. *Phys. Rev. B* **89**, 184413 (2014).

60. J. Krupka, Measurement of the complex permittivity, initial permeability, permeability tensor and ferromagnetic linewidth of gyromagnetic materials. *Meas. Sci. Technol.* **29**, 092001 (2018).

61. I. Syvorotka, H. Savytskyy, S. Ubizskii, A. Prabhakar, Growth and properties of sub-micrometer thin YIG-based LPE films using different fluxes. *Acta Phys. Pol. A* **141**, 261–267 (2022).

62. C. Dubs, O. Surzhenko, R. Linke, A. Danilewsky, U. Brückner, J. Dellith, Sub-micrometer yttrium iron garnet LPE films with low ferromagnetic resonance losses. *J. Phys. D Appl. Phys.* **50**, 204005 (2017).

63. G. Gurevich, A. N. Anisimov, Intrinsic spin wave relaxation processes in yttrium iron garnets. *Sov. Phys. JETP* **41**, 336–341 (1975).

64. G. A. Melkov, A. D. Dzyapko, A. V. Chumak, A. N. Slavin, Two-magnon relaxation reversal in ferrite spheres. *J. Exp. Theor. Phys.* **99**, 1193–1200 (2004).

65. A. G. Gurevich, G. A. Melkov, *Magnetization Oscillations and Waves* (CRC Press, ed. 1, 1996); https://taylorfrancis.com/books/9780429611278.

66. R. Erdélyi, G. Csaba, L. Maucha, F. Kohl, B. Heinz, J. Greil, M. Becherer, P. Pirro, Á. Papp, Design rules for low-insertion-loss magnonic transducers. *Sci. Rep.* **15**, 9806 (2025).

67. K. O. Levchenko, K. Davídková, J. Mikkelsen, A. V. Chumak, Review on spin-wave RF applications. *IEEE Trans. Magn.*, 1 (2026).

68. V. S. L'vov, A. Pomyalov, D. A. Bozhko, B. Hillebrands, A. A. Serga, Correlation-enhanced interaction of a Bose-Einstein condensate with parametric magnon pairs and virtual magnons. *Phys. Rev. Lett.* **131**, 156705 (2023).

69. F. Kühn, M. R. Schweizer, T. Azevedo, V. I. Vasyuchka, G. von Freymann, V. S. L'vov, B. Hillebrands, A. A. Serga, Enhancement of magnon flux toward a Bose-Einstein condensate. *Phys. Rev. B* **113**, 014409 (2026).

70. R. G. E. Morris, A. F. van Loo, S. Kosen, A. D. Karenowska, Strong coupling of magnons in a YIG sphere to photons in a planar superconducting resonator in the quantum limit. *Sci. Rep.* **7**, 11511 (2017).

71. E. Paladino, Y. M. Galperin, G. Falci, B. L. Altshuler, 1/f noise: Implications for solid-state quantum information. *Rev. Mod. Phys.* **86**, 361–418 (2014).

72. M. Pfirrmann, I. Boventer, A. Schneider, T. Wolz, M. Kläui, A. V. Ustinov, M. Weides, Magnons at low excitations: Observation of incoherent coupling to a bath of two-level systems. *Phys. Rev. Res.* **1**, 032023 (2019).

73. T. Wolz, L. McLellan, A. Schneider, A. Stehli, J. D. Brehm, H. Rotzinger, A. V. Ustinov, M. Weides, Frequency fluctuations of ferromagnetic resonances at millikelvin temperatures. *Appl. Phys. Lett.* **119**, 212403 (2021).

74. K. D. Crowley, R. A. McLellan, A. Dutta, N. Shumiya, A. P. M. Place, X. H. Le, Y. Gang, T. Madhavan, M. P. Bland, R. Chang, N. Khedkar, Y. C. Feng, E. A. Umbarkar, X. Gui, L. V. H. Rodgers, Y. Jia, M. M. Feldman, S. A. Lyon, M. Liu, R. J. Cava, A. A. Houck, N. P. de Leon, Disentangling losses in tantalum superconducting circuits. *Phys. Rev. X* **13**, 041005 (2023).

75. S. Kosen, H.-X. Li, M. Rommel, D. Shiri, C. Warren, L. Grönberg, J. Salonen, T. Abad, J. Biznárová, M. Caputo, L. Chen, K. Grigoras, G. Johansson, A. F. Kockum, C. Križan, D. P. Lozano, G. J. Norris, A. Osman, J. Fernández-Pendás, A. Ronzani, A. F. Roudsari, S. Simbierowicz, G. Tancredi, A. Wallraff, C. Eichler, J. Govenius, J. Bylander, Building blocks of a flip-chip integrated superconducting quantum processor. *Quantum Sci. Technol.* **7**, 035018 (2022).

76. Y. Tabuchi, S. Ishino, T. Ishikawa, R. Yamazaki, K. Usami, Y. Nakamura, Hybridizing ferromagnetic magnons and microwave photons in the quantum limit. *Phys. Rev. Lett.* **113**, 083603 (2014).

77. D. Lachance-Quirion, Y. Tabuchi, S. Ishino, A. Noguchi, T. Ishikawa, R. Yamazaki, Y. Nakamura, Resolving quanta of collective spin excitations in a millimeter-sized ferromagnet. *Sci. Adv.* **3**, e1603150 (2017).

78. J. Majer, J. M. Chow, J. M. Gambetta, J. Koch, B. R. Johnson, J. A. Schreier, L. Frunzio, D. I. Schuster, A. A. Houck, A. Wallraff, A. Blais, M. H. Devoret, S. M. Girvin, R. J. Schoelkopf, Coupling superconducting qubits via a cavity bus. *Nature* **449**, 443–447 (2007).

79. M. Kounalakis, S. Viola Kusminskiy, Y. M. Blanter, Engineering entangled coherent states of magnons and phonons via a transmon qubit. *Phys. Rev. B* **108**, 224416 (2023).

80. D. Awschalom, H. Bernien, R. Brown, A. Clerk, E. Chitambar, A. Dibos, J. Dionne, M. Eriksson, B. Fefferman, G. Fuchs, J. Gambetta, E. Goldschmidt, S. Guha, F. Heremans, K. Irwin, A. Jayich, L. Jiang, J. Karsch, M. Kasevich, S. Kolkowitz, P. Kwiat, T. Ladd, J. Lowell, D. Maslov, N. Mason, A. Matsuura, R. McDermott, R. van Meter, A. Miller, J. Orcutt, M. Saffman, M. Schleier-Smith, M. Singh, P. Smith, M. Suchara, F. Toudeh-Fallah, M. Turlington, B. Woods, T. Zhong, "A Roadmap for Quantum Interconnects" [Argonne, IL (United States), 2022]; https://doi.org/10.2172/1900586.

81. A. Megrant, Y. Chen, Scaling up superconducting quantum computers. *Nat. Electron.* **8**, 549–551 (2025).

82. P. Hansen, W. Tolksdorf, J. Schuldt, Anisotropy and magnetostriction of germanium-substituted yttrium iron garnet. *J. Appl. Phys.* **43**, 4740–4746 (1972).

83. J. F. Dillon, J. W. Nielsen, Effects of rare earth impurities on ferrimagnetic resonance in yttrium iron garnet. *Phys. Rev. Lett.* **3**, 30–31 (1959).

84. E. G. Spencer, R. C. LeCraw, R. C. Linares, Low-temperature ferromagnetic relaxation in yttrium iron garnet. *Phys. Rev.* **123**, 1937–1938 (1961).

85. E. G. Spencer, R. C. LeCraw, A. M. Clogston, Low-temperature line-width maximum in yttrium iron garnet. *Phys. Rev. Lett.* **3**, 32–33 (1959).

86. L. Mihalceanu, D. A. Bozhko, V. I. Vasyuchka, A. A. Serga, B. Hillebrands, A. Pomyalov, V. S. L'vov, V. S. Tyberkevych, Magnon Bose-Einstein condensate and supercurrents over a wide temperature range. Ukrainian. *J. Phys.* **64**, 927–927 (2019).

87. S. Wittekoek, T. J. A. Popma, J. M. Robertson, P. F. Bongers, Magneto-optic spectra and the dielectric tensor elements of bismuth-substituted iron garnets at photon energies between 2.2-5.2 eV. *Phys. Rev. B* **12**, 2777–2788 (1975).

88. M. Sparks, R. Loudon, C. Kittel, Ferromagnetic relaxation. I. Theory of the relaxation of the uniform precession and the degenerate spectrum in insulators at low temperatures. *Phys. Rev.* **122**, 791–803 (1961).

89. P. Pincus, M. Sparks, R. C. LeCraw, Ferromagnetic Relaxation. II. The role of four-magnon processes in relaxing the magnetization in ferromagnetic insulators. *Phys. Rev.* **124**, 1015–1018 (1961).

90. R. M. White, M. Sparks, Ferromagnetic relaxation. III. Theory of instabilities. *Phys. Rev.* **130**, 632–638 (1963).

91. S. Streib, N. Vidal-Silva, K. Shen, G. E. W. Bauer, Magnon-phonon interactions in magnetic insulators. *Phys. Rev. B* **99**, 184442 (2019).

92. T. Kasuya, R. C. LeCraw, Relaxation mechanisms in ferromagnetic resonance. *Phys. Rev. Lett.* **6**, 223–225 (1961).

93. D. I. Tchernev, Effect of low-temperature magnetic anneal on the linewidth of garnets containing Fe2+ ions. *J. Appl. Phys.* **38**, 1046–1047 (1967).
